# Differential and Synergistic Functionality of Acylsugars in Suppressing Oviposition by Insect Herbivores

**DOI:** 10.1371/journal.pone.0153345

**Published:** 2016-04-11

**Authors:** Brian M. Leckie, Damon A. D'Ambrosio, Thomas M. Chappell, Rayko Halitschke, Darlene M. De Jong, André Kessler, George G. Kennedy, Martha A. Mutschler

**Affiliations:** 1 Section of Plant Breeding and Genetics, School of Integrative Plant Science, Cornell University, Ithaca, New York, United States of America; 2 Department of Entomology, North Carolina State University, Raleigh, North Carolina, United States of America; 3 Department of Ecology and Evolutionary Biology, Cornell University, Ithaca, New York, United States of America; Zhejiang University, CHINA

## Abstract

Acylsugars are secondary metabolites exuded from type IV glandular trichomes that provide broad-spectrum insect suppression for *Solanum pennellii* Correll, a wild relative of cultivated tomato. Acylsugars produced by different *S*. *pennellii* accessions vary by sugar moieties (glucose or sucrose) and fatty acid side chains (lengths and branching patterns). Our objective was to determine which acylsugar compositions more effectively suppressed oviposition of the whitefly *Bemisia tabaci* (Gennadius) (Middle East—Asia Minor 1 Group), tobacco thrips, *Frankliniella fusca* (Hinds), and western flower thrips, *Frankliniella occidentalis* (Pergande). We extracted and characterized acylsugars from four *S*. *pennellii* accessions with different compositions, as well as from an acylsugar-producing tomato breeding line. We also fractionated the acylsugars of one *S*. *pennellii* accession to examine the effects of its components. Effects of acylsugars on oviposition were evaluated by administering a range of doses to oviposition sites of adult whiteflies and thrips in non-choice and choice bioassays, respectively. The acylsugars from *S*. *pennellii* accessions and the tomato breeding line demonstrated differential functionality in their ability to alter the distribution of whitefly oviposition and suppress oviposition on acylsugar treated substrates. Tobacco thrips were sensitive to all compositions while western flower thrips and whiteflies were more sensitive to acylsugars from a subset of *S*. *pennellii* accessions. It follows that acylsugars could thus mediate plant-enemy interactions in such a way as to affect evolution of host specialization, resistance specificity, and potentially host differentiation or local adaptation. The acylsugars from *S*. *pennellii* LA1376 were separated by polarity into two fractions that differed sharply for their sugar moieties and fatty acid side chains. These fractions had different efficacies, with neither having activity approaching that of the original exudate. When these two fractions were recombined, the effect on both whiteflies and thrips exceeded the sum of the two fractions’ effects, and was similar to that of the original exudate. These results suggest that increasing diversity of components within a mixture may increase suppression through synergistic interactions. This study demonstrates the potential for composition-specific deployment of acylsugars for herbivore oviposition suppression, either through *in planta* production by tomato lines, or as biocides applied by a foliar spray.

## Introduction

Plants produce an enormous diversity of secondary metabolites with specialized functions [1,2]. These compounds exist in a wide range of classes with vast numbers of smaller modifications and serve vital roles in plant-environment interactions, particularly in plant defense where they can act as antagonists to insect herbivores [3]. Understanding the structure-function relationships of this in-class and overall structural diversity of secondary metabolites is necessary to assess the role of these compounds in mediating species interactions and to utilize them in pest control. Improving plants to optimize their defensive secondary metabolite production for resistance to targeted specific pests could provide a strong tool for sustainable pest control in agriculture and agricultural ecosystems; this can most efficiently be achieved when key components underlying defensive efficacy and their interactions have been identified.

Levels of defensive secondary metabolites are thought to have been reduced as a result of domestication in several important crop species [4–6]. One such plant is the cultivated tomato [7], *Solanum lycopersicum*, a vegetable of world-wide economic importance, thought to have been domesticated from its weedy relative *S*. *lycopersicum* var *cerasiforme* [8]. Cultivated tomatoes are attacked by an estimated 100–200 insect species and include members from many insect orders including major pests in the orders Lepidoptera, Hemiptera, and Thysanoptera [9]. The latter two of these orders contain vectors of major tomato viruses. *Bemisia tabaci* (Gennadius) (Hemiptera: Aleyrodidae) Middle East—Asia Minor 1 Group (MEAM1), causes direct damage to tomato through its saliva’s toxic effects and plant debilitation from sap removal [10]and indirectly as it is the vector of *tomato yellow leaf curl virus*, which is capable of causing severe stunting, reduced leaf size, chlorosis, and premature flower drop. Infection early during cultivation can result in 100% yield loss [11–13]. The tobacco thrips, *Frankliniella fusca* (Hinds) (Thysanoptera: Thripidae) and the western flower thrips, *F*. *occidentalis* (Pergande) (Thysanoptera: Thripidae) are important pests of tomato, causing direct feeding damage that can stunt young plants, but are more important as vectors of *tomato spotted wilt virus* (TSWV). The susceptibility of tomatoes to direct and indirect damage caused by insect herbivores, and to diseases vectored by insects, may be due in part to reduced production of insect-affecting secondary metabolites. Breeding defensive secondary metabolic production into cultivated tomato may supplement current defenses, and provide a durable means of controlling insect-mediated damage to plants.

Acylsugars are one of the most promising classes of plant-derived control agents associated with insect resistance and are produced by a diversity of taxa in the Solanaceae; including some species in the *Nicotiana*, *Solanum*, *Petunia*, and *Datura* genera [14–28]. *Solanum pennellii* (Correll) D’Arcy accession LA716, a wild relative of tomato, is a valuable breeding source for the improvement of cultivated tomato for insect resistance as it produces high levels of multiple acylsugars that are exuded in droplets at the tips of type IV glandular trichomes present on all green parts of the plant. Acylsugars are sugar esters each comprising a base moiety of either glucose or sucrose with 3–4 fatty acid side chains that vary in branching patterns and length. The acylsugars of *S*. *pennellii* LA716 consist primarily of acylglucoses with 2-methylpropanoic acid (i-C4) and 8-methylnonanoic acid (i-C10) as the primary fatty acid side chains [29,30]. The acylsugars of LA716 have been shown to be effective in mediating plant resistance to a wide range of insects [19,20,22,23] including *B*. *tabaci* [24].

The native range of *S*. *pennellii* is a narrow stretch of the western slopes of the Peruvian Andes with accessions collected from sea level to elevations over 1900 m [30]. Significant variations exist in the acylsugar compositions of these accessions; with southern accessions producing almost completely acylglucoses with primarily i-C4 fatty acids, while northern accessions produce a mixture of acylglucoses and acylsucroses and primarily 3-methylbutanoic (i-C5) fatty acid. The geographic distribution of dominant chemotypes can be a result of genetic drift or local adaptation but provides spatially differential challenges for interacting antagonists, such as herbivores and pathogens [31]. Assuming natural selection as the generator of geographic structure, the different compositions may reflect the multi-functionality of acylsugars and thus a potential optimization of abundance and composition of the acylsugar bouquet to varying environmental conditions. In the same way this chemotype diversity provides the substrate for natural selection to act on; it provides an excellent tool for chemical structure-function analyses and a basis for breeding tomatoes for increased resistance [32] or for their potential use as foliar applied crop protection chemicals.

The Cornell University tomato breeding program crossed *S*. *pennellii* LA716 with cultivated tomato to then derive tomato breeding lines with acylsugar-mediated insect resistance. The benchmark acylsugar breeding line, CU071026, produces ~15% of the acylsugar levels of LA716 but with a different composition [32–34]. This line shows effective control of *B*. *tabaci* in field cage trials under heavy infestation [32]. Multiple QTL underlying sugar moiety as well as fatty acid profile have been identified from a BC_1_F_1_ population (*S*. *pennellii* LA716 x CU071026) x CU071026) and from a intraspecific *S*. *pennellii* BC_1_F_1_ population allowing manipulation of the acylsugar component parts [33–36].

The objective of this study was to determine which compositions or key components of acylsugars were more effective at suppressing oviposition of three insect herbivores on acylsugar-treated substrates. We tested multiple rates of purified acylsugar extracts from four *S*. *pennellii* accessions and the acylsugar breeding line CU071026, differing for both acylsugar fatty acid profiles and base moieties, in bioassays to evaluate their impacts on oviposition. One acylsugar extract was further fractionated and tested to evaluate efficacy of fractions with different base moieties compared to a reconstituted blend of the fractions. Results indicate that the effects of different acylsugar compositions are concentration dependent and affect insect species differently, and that synergism underlies the net effect of acylsugar mixtures produced by the plants.

## Materials and Methods

### Plant materials and their Characterization

Seeds from *S*. *pennellii* accessions LA1732, LA1376, and LA2560 were obtained from the Tomato Genetics Resource Center (TGRC) at the University of California at Davis. Seeds from *S*. *pennellii* LA716 were produced by the Cornell University tomato breeding program, derived from seed originally obtained from the TGRC. CU071026 is an acylsugar-producing tomato line produced by the Cornell University tomato breeding program; *S*. *pennellii* LA716 was a parent in the pedigree of CU071026 [32]. All plant entries were grown in the Guterman Bioclimatic Laboratory and Greenhouse Complex at Cornell University in Ithaca NY and maintained at 29°C with a 16:8 h light:dark photoperiod.

All *S*. *pennellii* and CU071026 plants were individually tested at 9 weeks after sowing for acylsugar level, acylglucose production, and acylsucrose production by methods described in Leckie *et al*. [33]. Acylsugar fatty acids for all plants were characterized by GC-MS by methods described in Leckie *et al*. [34] to identify plants, if any, that vary in composition within accessions. As a results, three plants of accession LA1376 and six plants of LA2560 had off-type fatty acid profiles and were discarded. The remaining 8 to 15 plants per accession were grown for an additional three months, then all leaves and stems were then collected in bulk within accession and dried to completion in a seed dryer (30°C) for use in acylsugar isolation.

### Isolation, purification and characterization of acylsugars

Dried tissue from each accession and CU071026 was washed briefly in methanol to remove acylsugars. Rinsates were passed through a coarse wire mesh to remove larger pieces of debris, then passed through a P5 qualitative filter paper (Cat # 09-801C, Fisher Scientific) in a Buchner-type conical funnel to remove fine particulate. Acylsugars were concentrated by evaporation, then were purified using a dry column vacuum chromatography protocol derived from that of Pedersen and Rosenbohm [37]. Approximately 12 grams of concentrated acylsugars from each accession were diluted with methylene chloride and combined with 15–40 μM silica gel (Cat # 15111–3, EMD Chemicals) at a weight to weight ratio of 2:3 acylsugar to silica gel. The combination was dried in a glass petri dish. A 600 ml fritted funnel (Cat # 36060, Pyrex) was packed with 5.5 cm of 15–40 μM silica gel (Cat # 15111–3, EMD Chemicals). Packed columns were placed on a filter flask attached to a vacuum. Dried acylsugar silica suspensions were then layered on top of a P5 qualitative filter paper (Cat # 09-801C, Fisher Scientific) cut to fit into the funnel. An additional filter paper was placed on top of the sample to reduce sample disruption when washing the column. Columns were washed under vacuum pressure four times with 500 ml of methylene chloride containing 3% acetone. Acylsugars were then recovered from the column with two 500 ml acetone washes. Excess acetone was removed by evaporation.

Acylsugars of *S*. *pennellii* LA1376, previously identified as producing both acylsucroses and acylglucoses [30], were fractionated into acylsucrose and acylglucose fractions using a modified protocol with five 500 ml recovery washes of increasing polarity (Methylene chloride containing 20, 40, 60, 80, and 100% acetone). Fractions collected at 20 and 40% were combined to form the less polar fraction (Fr-LP) while the 80 and 100% fractions were combined to form the more polar fraction (Fr-MP). The 60% fraction was not included as it contained only trace levels of both acylsucroses and acylglucoses. Purified acylsugars and fractions were characterized for acylsugar base moiety and fatty acid profiles using the methods described above [33,34] and analyzed for background phenolic contaminants using methods in S1 Text.

### Whitefly and Thrips colonies

*B*. *tabaci* MEAM1, as determined by Dr. Cindy McKenzie (USDA-ARS) using PCR analysis with primers based on the mtCOI gene [38–41], were maintained on the tomato line NC123S [42] in multiple cages (1462W, Bioquip Products Inc.) within a climate controlled growth chamber in the Guterman Bioclimatic Laboratory and Greenhouse Complex at Cornell University in Ithaca, NY. Whiteflies used to start this colony were provided by Dr. John Sanderson, Department of Entomology at Cornell University.

Adult, 14–15 day old female *F*. *fusca* and *F*. *occidentalis* for all thrips experiments were obtained from laboratory colonies. Each of these colonies was maintained separately and reared on *Phaseolus vulgaris* L. bean pods in a controlled environment of 26°C with ca. 60% RH. *F*. *fusca* were reared in a photoperiod of 14:10, and *F*. *occidentalis* were reared in constant light. Rearing conditions for each species were those that resulted in the most rapid colony growth and were selected to ensure that consistently high reproductive output so that sufficient thrips were available when needed for our experiments.

### Overview of Bioassay Objectives

A series of bioassays was performed to determine the relative activities of the purified acylsugar extracts on oviposition of the three insect species. Initial assays were conducted to evaluate the effect of five purified acylsugars (LA716, LA1376, LA1732, LA2560, and CU071026) to determine if acylsugar composition is important in suppressing whitefly and thrips oviposition. Further evaluations were performed on the fractions (Fr-MP and Fr-LP) of the LA1376 acylsugars and a 1:1 blend of the fractions (Fr-MP/Fr-LP) to determine if one of these fractions was more effective at suppressing oviposition, and if the 1:1 blend was as effective as the original LA1376 acylsugar extract.

#### Whitefly bioassays

A series of oviposition bioassays was performed to test the response of *B*. *tabaci* (MEAM1) to the purified acylsugars. Bioassays were performed over time with multiple, randomized acylsugar chemistries tested during any given experiment, and the number of chemistries tested being limited by logistical constraint. Each purified acylsugar extract was tested in at least two experiments on different days. Purified acylsugar extracts were tested at five rates (1, 7, 13, 19, and 25 mg/mL). Each administered extract-rate combination was replicated three times on separate leaf discs.

All whitefly bioassays were conducted in a climate-controlled, walk-in growth chamber in the Guterman Bioclimatic Laboratory and Greenhouse Complex at Cornell University in Ithaca, NY. The tomato line NC123s was grown at 25°C under a 16:8 h light:dark photoperiod in a growth chamber to provide leaf tissue for bioassays. Five weeks after sowing the terminal laterals from the fourth leaf position were collected and leaf discs of diameter 2.6 cm were punched on both sides of the mid-vein with the open end of a 50 ml Falcon Tube. Leaf discs were positioned 16 cm under an artist's airbrush (Type H, Paasche airbrush, Chicago, IL) pointing down and held in position with a clamp on a ring stand. The air for the brush was supplied by a compressor equipped with a regulator (D220R, Paasche airbrush, Chicago, IL) set at 207 kPa. Purified acylsugars dissolved in 95% Ethanol at rates of 0.33, 2.33, 4.33, 6.33, and 8.33 mg/ml or a 95% ethanol control were applied to the abaxial side of the leaf disc in three sequential 50 μl applications. Sequential applications were performed to provide more uniform application of acylsugars to the leaf surface. To maintain freshness, each sprayed leaf disc was placed, abaxial side up, onto a thin layer of 0.75% water agar paste covering a 7 mm firm layer of 1.5% water agar media in a 35 x 10 mm petri dish. Petri dishes were then placed into round plastic containers (473 ml) (PK16S, Fabri-Kal Corp) used as bioassay arenas by attaching the bottom of each open petri dish containing a sprayed leaf disc to the inside center of a plastic arena lid (9505261, Fabri-Kal Corp). Design of bioassay arenas was based on those by Firdaus et al [43]. The plastic containers were equipped with two side air vents of approximately 3 cm in diameter covered with mesh screens (7260B, Bioquip Products Inc.). Adult whiteflies from a four-day-old cohort were collected by luring them to a yellow surface placed in the bottom of a petri dish, cooled to immobilize, shaken on to a cold steel plate, and loaded into the testing arenas. Approximately 20 adults were gently added to each plastic container from the metal plate by blowing a stream of air through a glass pipette tip. Containers were sealed with the lids on the top and placed into two large plastic boxes with free water coating the bottom to provide humidity. Boxes were maintained at 25°C for three days with a 16:8 h light:dark photoperiod in a walk-in growth chamber to maximize oviposition while not jeopardizing leaf freshness [43]. After 3 days, data were collected on total numbers and sexes of adults, numbers and sexes of living adults, and number of eggs. Because prior work [32] showed that there might be differences in egg distribution due to the presence of acylsugars, data were also collected for the number of egg clusters, as well as numbers of eggs per cluster. Egg clusters were defined as three or more eggs oviposited in close proximity and spaced no more than two egg widths from each other. Eggs within a cluster may have been laid by a single female in one or more oviposition events or by multiple females. Dead and immobilized whiteflies incapable of ovipositing were counted as half of an individual as they are assumed to have persisted or been motile for an average of half the testing interval.

#### Thrips bioassays

A similar series of bioassays was performed to test oviposition response of the two thrips species to the purified acylsugars and fractions of LA1376. Again, Bioassays were performed over time with multiple randomized acylsugar chemistries tested during each of four experiments on different days. Extracts were tested at a total of eleven rates (0, 0.5, 1, 2, 4, 5, 6, 8, 10, 15, 20 mg/mL) applied in 95% ethanol. Each administered extract-rate combination was replicated six times in separate bioassay tubes. The bioassay procedure used to quantify the effect of acylsugar extracts on oviposition by *F*. *fusca* and *F*. *occidentalis* was modified from that of [44]. The resulting assay observes individual eggs laid on eligible surfaces on opposing sides of an assay chamber, such that the response metric is a probability of a given egg being laid on one *vs*. the other surface. This element of choice in the assay allows us to marginalize aggregate oviposition variation across insects in assays; a given insect may oviposit few or many times, and the effect of acylsugar in either situation is the same if the ratio of oviposition on treated *vs*. untreated surfaces is consistent. Assay chambers were constructed of 55 mm sections of extruded acrylic tubing with an inner diameter of 9.5 mm and an outer diameter of 12.7 mm (U.S. Plastic Corp., Lima, OH, USA).

Oviposition substrates were constructed by removing the caps from 1.5 mL polypropylene microcentrifuge tubes (ThermoFisher Scientific, Waltham, MA, USA) and filling the caps with 155 μL of 3% sucrose solution containing 0.616% (616μL/100mL) green food color (McCormick & Co., Inc., Hunt Valley, MD, USA). 15 mm x 15 mm squares of Parafilm (Bemis Flexible Packaging, Neenah, WI, USA) were stretched to approximately twice their length in each dimension and placed atop the fluid filled caps, creating a watertight membrane that could readily be penetrated by thrips mouthparts and ovipositors.

Each bioassay chamber consisted of the acrylic tube capped on each end with an oviposition substrate, one of which was surface-treated with acylsugar extract, while the other received no treatment. Assays containing a surface treated at a rate of 0 mg/mL served as controls. Acylsugar treatments were applied as described for the *B*. *tabaci* bioassays. 40 mg/mL acylsugar stock solutions were prepared in 95% ethanol at the beginning of the day they were to be used and diluted with 95% ethanol to produce the desired concentrations of acylsugars for application. Solutions were further diluted by 50% and delivered to the oviposition substrate in two successive 50 μL spray bursts to ensure more uniform dispersal on the oviposition substrate.

All thrips experiments took place under constant light provided by two 40-watt fluorescent plant and aquarium bulbs (GE Lighting, Cleveland, OH, USA) suspended 30 cm above the assays. To account for possible phototaxis as a nuisance effect in these assays, the orientation of assay tubes was manipulated: 3 groups of assay tubes were randomly selected, and the assays in these groups were rotated 180° so that the surface-treated oviposition substrates in these blocks were oriented towards the left side of the light source whereas the surface-treated substrates in the other 3 blocks remained oriented towards the right side. Locations of treatments within each group as well as directional orientation of groups were re-randomized for each experiment.

Bioassays were initiated by placing 0.3 mg of pine pollen at the midpoint of the tube and adding a single adult female thrips. After the introduction of the thrips, both ends of the tube were immediately capped with the oviposition substrates. After 60 hours, the assays were disassembled. The number of eggs on each oviposition substrate was recorded. All eggs were oviposited on an oviposition substrate; no eggs were oviposited on the interior assay tube surface. Each counted egg was treated as one observed choice made by an ovipositing female, and this binary choice response was analyzed as a logistic regression.

### Data Analysis

#### Whitefly data

Data from whitefly bioassays were analyzed to compare the impacts of specific rates and acylsugar treatment combinations. The oviposition response metrics analyzed were the relative oviposition sites per female (ROSPF), relative total numbers of eggs per female (REPF), the proportion of eggs oviposited as singletons, and the total number of eggs oviposited including singletons and those in clusters. The proportion of singletons oviposited was calculated as

proportion singletons=SE/(SE+CE) (1)

ROSPF and REPF were calculated as

ROSF=[(SE+CL)/(L+0.5*D)]/ROSF control (2)

REPF=[(SE+CE)/(L+0.5*D)]/REPF control (3)

where SE = eggs laid singly on leaf disc, CL = number of clusters on leaf disc, CE = number of eggs in clusters on leaf disc, L = living females at the end of the experiment, D = dead females at the end of the experiment, and ROSPF_control_ and REPF_control_ were calculated as

ROSFcontrol=(SE+CL)/(L+0.5*D) (4)

REPFcontrol=(SE+CE)/(L+0.5*D) (5)

for those observations in which no acylsugar treatment was administered. In other words, ROSPF and REPF normalize oviposition of whiteflies to an expected rate of oviposition in the absence of treatment, such that proportional reduction in oviposition can be meaningfully described. Because ROSPF and REPF represent proportions varying continuously between zero and one, a beta response distribution and logit link function were used in model fitting. Models were fitted for each acylsugar extract using the GLIMMIX procedure of the SAS system (version 9.4, SAS Institute, Cary, NC), with date-of-assay included as a random effect, and acylsugar application rate in mg/mL as a fixed effect.

#### Thrips data

Choice assays were first analyzed to test for possible influence of egg density on a female’s choice, using the GLIMMIX procedure of the SAS system (version 9.4, SAS Institute, Cary, NC). A binary response distribution and logit link function were specified, and maximum likelihood was used for fitting. Independent variables treated as fixed effects in this analysis included the total number of eggs laid by a female during an assay (the sum of those laid on each of the two surfaces), the rate at which a given compound was applied to the treated side of the assay tube, and the interaction between these two effects. No effect of phototaxis was observed. We thereby excluded light orientation group membership from analysis.

Secondly, a subset of choice assays in which thrips were offered either two identical acylsugar-treated or 95% ethanol-treated oviposition surfaces were analyzed to confirm that females were making a choice by interact with both sides of assay tubes during experiments. Eggs within tubes were counted at 24 and 48 hours after beginning choice assays. Independent variables treated as fixed effects in this analysis included the amount of time elapsed since beginning the assay, the rate at which a given compound was applied to the treated side of the assay tube, and the interaction between these two effects. Because no significant effect of elapsed time or its interaction with rate was found, oviposition was understood to occur at a rate independent of assay time, and subsequent analyses involved counting eggs at the end of bioassays.

Another check for experimental robustness involved examining the possibility that the 95% ethanol carrier might itself have an effect on oviposition. To test for this effect, a sham application of only 95% ethanol was administered to one surface of a bioassay tube, while the other surface was left untreated. No effect of this sham application was detected (*F*_*1*,*540*_ = 1.52, *p* > 0.2) using ANOVA (the GLM procedure of the SAS system), in which number of eggs was analyzed as a function of treatment (sham or none) and assay tube (with one insect per tube). Accordingly, analysis proceeded as described below.

Dose response curves were fitted separately for each compound and thrips species. Using the LOGISTIC procedure of the SAS system, specifying a logit link function and no-intercept model, choice response was modeled as a function of the rate of compound application on treated sides of assay tubes. Effective concentrations for given probabilities of eggs’ being laid on treated surfaces (EC_p_) were calculated as follows:

ECp=log(p/(1−p))/β (6)

where *p* is the probability of an egg’s being laid on the treated surface of an assay tube, and β is the regression coefficient from the logistic regression of choice response on compound application rate. 95% confidence intervals around EC_p_ point estimates were calculated by using upper and lower 95% confidence limits for respective coefficients β in the equation above. Proportional decrease in the probability *p* per unit increase in compound application rate was calculated by exponentiating the coefficient β for each analysis.

#### Testing for synergistic response to extract fractions

Two fractions (Fr-MP and Fr-LP) of the *S*. *pennellii* LA1376 extract were tested separately and when recombined in a 1:1 mixture for activity against *B*. *tabaci* (MEAM1) and *F*. *occidentalis* using the previously described bioassays. For whitefly bioassays, individual fractions and the mixture were tested at all rates as previously described. For thrips assays, each fraction was tested at rates of 3 mg/mL and 6 mg/mL. A 1:1 mixture of the two fractions was created by combining equal parts of both fraction solutions at 6 mg/mL, thereby creating a mixture with an overall acylsugar concentration of 6 mg/mL wherein each fraction was represented at 3 mg/mL upon application. Data were analyzed to test for effects of mixture component rates and their interaction on the oviposition response variables, by fitting a generalized linear model as described in the “whitefly data” section, but including the two input variables Fr-MP rate and Fr-LP rate, and their two-way interaction. Synergism or antagonism between the two components would be indicated by a significant interaction term in the model fit.

## Results and Discussion

### Characterization of acylsugars for use in whitefly and thrips assays

The acylsugar samples used in the whitefly and thrips bioassays were isolated from the acylsugar-producing tomato breeding line CU071026 and from four *S*. *pennellii* accessions, chosen based on prior acylsugar characterization [30] and purified. HPLC analysis of initial and purified plant extracts identified the effective removal of 95% of metabolites other than acylsugars (S1–S7 Figs). Extracts were then characterized to confirm the identities of the sugar and fatty acid substituents of each acylsugar sample. This characterization of the purified acylsugars revealed fatty acid and base moiety profiles (Table 1) similar to those previously reported for these plant sources [30,34]. GC-MS characterization and analysis of sugar moieties of the acylsugars of CU071026 identified only acylsucroses with ai-C5 (9.2%), i-C5 (76.4%) and n-C12 (10.0%) as the predominant esterified fatty acid side chains. In a previous study these same acids were detected as the primary side-chains of CU071026, although their percentages of the total were different at 18.0, 41.8, and 38.2%, respectively [34]. These differences could be due to environmental changes between the growing conditions or the result of purification of acylsugars from the entire plant instead of just leaf tissue. Previous studies have shown that the acylsugars produced at trace levels by the tomato M82 are also acylsucroses with similar fatty acids, although there were slight differences in the relative levels of ai-C5 (~30%), i-C5 (~57%), and n-C12 (~7%) [45]. Characterization of the purified extracts from the four *S*. *pennellii* accessions revealed contrasting acylsugar profiles. The two accessions LA1732 and LA716 both accumulated almost exclusively acylglucoses and high amounts of i-C4 fatty acids, while the two accessions LA2560 and LA1376 accumulated a mixture of acylglucoses and acylsucroses with high levels of i-C5 fatty acid side chains. Correspondence between these two groups and their geographic occurrence confirms that the acylsugar composition may relate to geography, as previously reported [30]. LA1732 and LA716 occur in the northern natural range of *S*. *pennellii*; LA2560 and LA1376 occur in the southern range. Variation in length of predominant extended iso-even fatty acids was also observed: for example, LA1732 and LA716 accumulated i-C8 and i-C10, respectively. Further variation among acylsugar profiles are summarized in (Table 1), and the extent of this variation provided the basis for studying potentially differential functionality of acylsugar compositions.

**Table 1:** Characterization of acylsugar compositions (base moiety and fatty acids) from *Solanum pennellii* accessions, CU071026 (an acylsugar tomato breeding line), the less polar LA1376 fraction (Fr-LP), the more polar LA1376 fraction (Fr-MP), and the 1:1 mixture of these fractions (Fr-MP/Fr-LP).

Acylsugar source	Type	%AG^b^	% Total Fatty Acids^a^										
			i-C4	ai-C5	i-C5	ai-C6	i-C8	n-C8	i-C9	i-C10	n-C10	i-C11	n-C12
CU071026	AS-line	1.5	3.1	9.2	76.4	0.4	0.0	0.0	0.0	0.0	0.6	0.0	10.0
LA716	Southern	94.7	75.4	4.5	0.8	0.0	0.0	0.0	0.0	13.6	4.2	0.1	1.2
LA1732	Southern	95.8	75.1	6.0	0.7	0.1	16.0	1.3	0.1	0.2	0.1	0.0	0.0
LA2560	Northern	55.6	2.5	3.0	80.9	5.3	0.0	0.0	0.7	0.3	0.8	1.3	5.0
LA1376	Northern	67.3	4.1	3.8	64.4	11.8	0.0	0.4	0.9	0.2	6.3	1.8	5.7
Fr-LP	Fraction	100.0	4.0	5.4	67.2	11.7	0.0	0.4	0.9	0.1	4.6	1.2	3.8
Fr-MP	Fraction	0.0	6.5	6.5	23.3	7.1	0.0	0.3	1.3	0.4	18.0	6.6	27.7
Fr-MP/Fr-LP	Mixture	50.0	3.8	3.3	58.1	9.6	0.0	0.3	0.9	0.2	8.7	2.8	11.3

^a^ Individual fatty acids with no values over 0.5% were considered trace and removed from table.

^b^Percent Acylglucoses

### Fractionation of LA1376 Acylsugars

We separated the purified acylsugars of *S*. *pennellii* LA1376 by dry column vacuum chromatography into two fractions, the less polar fraction (Fr-LP), which was completely acylglucoses, and the more polar fraction (Fr-MP), which was acylsucroses (Table 1). GC-MS characterization of the Fr-LP revealed a fatty acid profile that was almost identical to the unfractionated LA1376 profile, while the Fr-MP was unique as more than 50% of its attached fatty acids were longer chain lengths (ten to twelve carbons) whereas all other extracts consisted primarily of short chain acids (four to five carbons). These results reveal that fatty acids are not attached to the different base moieties at the same frequencies and that some acids are preferentially attached to a specific moiety; implicating a possible role of acyltransferases with specific activity or preferential activity for the different base moieties. When combined in a 1:1 ratio by mass, the blend (Fr-MP/Fr-LP) contained a 1:1 mixture of acylsugar base moieties and a fatty acid profile similar to that of the unfractionated LA1376 profile but with slight increases in n-C10 and n-C12 acids. These fractions and their mixture provide acylsugar pools ideal for targeted testing of composition components responsible for the suppression activities of LA1376.

### Oviposition bioassays

Clusters of whitefly eggs were observed on acylsugar-treated leaf discs similar to those reported previously on foliage of acylsugar breeding lines [32], while on control leaf discs eggs were laid in a random distribution across the leaf discs, similar to eggs found on leaves of tomato controls in field trials. These clusters are the result of one or more whitefly ovipositing multiple eggs at a particular location. Hence, eggs in a cluster were considered to represent a single oviposition site.

Whiteflies exposed to control leaf discs on average laid high numbers of eggs per female (19.1) and had an average of 18.9 oviposition sites per female (S1 Table). Whiteflies exposed to leaf discs treated with acylsugar extracts exhibited a wide range in average eggs per females (3.6–17.8) and average oviposition sites per female (2.9–17.1). All purified acylsugar treatments extracted from *S*. *pennellii* accessions and from CU071026 decreased oviposition sites when compared to the control, but were not equally effective. At a rate of 25 mg/mL (the highest observed), the LA1376 acylsugars reduced oviposition by 85% to 2.9 oviposition sites per female.

**Fig 1:**
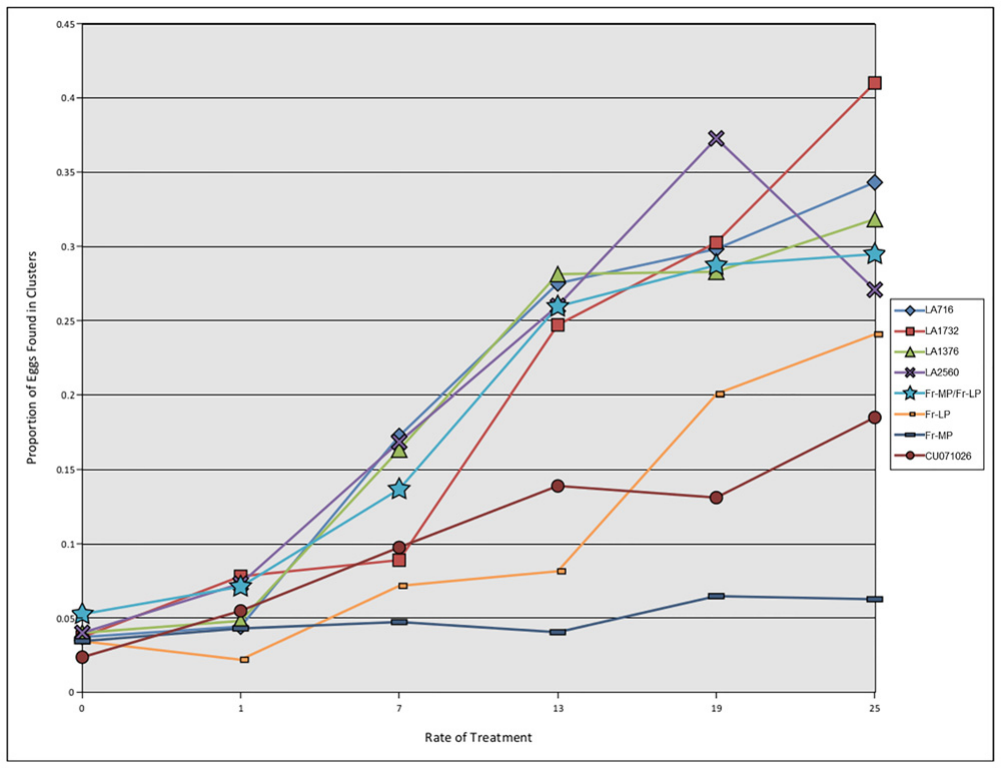
Proportion of Eggs laid in clusters by whiteflies presented leaf discs treated with increasing rates of different acylsugar extracts.

Dose response curves were fit for each compound, and effective concentrations required to reduce oviposition by 50% (EC50) were estimated (Table 2). The lowest efficacies in suppressing oviposition on treated foliage were observed with the acylsugars of CU071026, which consist largely of acylsucroses and has high levels of i-C5 fatty acids. Intermediate efficacies were observed with acylsugars from LA716 and LA1732, which are almost exclusively acylglucoses and have high levels of i-C4 fatty acids. The highest efficacies were observed from the acylsugars from LA2560 and LA1376, which contain a mixture of acylsugars with both base moieties (acylglucoses and acylsucroses) and/or high levels of i-C5 fatty acids. Analysis of total eggs (Table 2 and S1 Table) was also performed and produced results highly consistent with oviposition site analysis. These results lead to hypotheses that either a blend of the two moieties might be important for suppression of oviposition, or that fatty acid profiles may be of greater relative importance than sugar moiety for the insects’ oviposition. Extracts altered oviposition behavior resulting in increased clustering of the eggs (Fig 1) and a reduced number of eggs (S8 Fig) laid on acylsugar-treated foliage in a manner that reflected both extract activity and concentration.

**Table 2:** Estimated rates in mg/mL required to reduce oviposition by 50% (EC50, “effective concentration” for 50% reduction) for each acylsugar source. Acylsugar sources include purified acylsugar extracts, *S*. *pennellii* LA1376 fractions, and a blend of fractions. The analyzed responses of *B*. *tabaci* (MEAM1) were ROSPF, the rates at which females changed oviposition sites relative to a control treatment; and REPF, the rates at which females oviposited eggs (aggregate number of eggs) relative to a control treatment, during non-choice leaflet oviposition bioassays. The analyzed response of *F*. *occidentalis* and *F*. *fusca* was the probability of oviposition to a treated synthetic membrane during choice bioassays.

Acylsugar source	*B*. *tabaci*		*F*. *occidentalis*	*F*. *fusca*
	EC50 for ROSPF and 95% confidence interval	EC50 for REPF and 95% confidence interval	EC50 and 95% confidence interval	EC50 and 95% confidence interval
CU071026	25.0 (18.4 / 38.7)	20.1 (13.6 / 39.2)	19.6 (15.5 / 26.7)	3.1 (2.8 / 3.5)
LA716	18.6 (15.0 / 24.7)	17.4 (12.6 / 28.0)	5.8 (5.3 / 6.4)	3.9 (3.5 / 4.6)
LA1732	16.0 (12.9 / 21.0)	9.8 (7.8 / 13.1)	4.8 (4.4 / 5.4)	3.3 (3.1 / 3.7)
LA2560	12.5 (10.1 / 16.3)	9.9 (7.7 / 14.1)	2.9 (2.6 / 3.2)	3.7 (3.3 / 4.3)
LA1376	11.0 (9.3 / 13.5)	7.4 (6.1 / 9.3)	3.1 (2.8 / 3.4)	2.2 (2.0 / 2.6)
Fr-LP	23.7 (18.7 / 32.5)	14.7 (10.5 / 24.4)	3.5 (3.1 / 4.1)	NT
Fr-MP	63.1 (33.5 / 555.5)	29.2 (16.5 / 126.3)	6.3 (5.3 / 7.9)	NT
Fr-MP/Fr-LP	12.5 (9.6 / 17.7)	10.5 (8.1 / 15)	3.4 (3.0 / 4.1)	NT

NT = Not Tested

Oviposition by two thrips species was evaluated using assays in which thrips were simultaneously presented with acylsugar-treated and untreated oviposition surfaces (S2 and S3 Tables). Dose response curves were fit for each compound and effective concentrations required to reduce probability of oviposition on treated surfaces by 50% (EC50) were estimated (Table 2). All purified acylsugar treatments reduced oviposition on the treated substrate by *F*. *occidentalis* performance. Similar to assays involving *B*. *tabaci*, the most effective acylsugar (LA2560) is that which contained the highest proportion of acylsucroses and i-C5 of all *S*. *pennellii* accessions, again suggesting that these compounds are important for suppressing oviposition. Assays involving *F*. *fusca* revealed a higher sensitivity to all purified acylsugar extract treatments than was observed for either *F*. *occidentalis* or *B*. *tabaci* (Table 2). *F*. *fusca* was observed to have noteworthy sensitivity to CU071026, unlike *F*. *occidentalis* or *B*. *tabaci* to this extract.

To compare the efficacies of the extracts, rates of proportional reduction in oviposition on treated surfaces were calculated for each insect species-extract combination. This calculation provides a simple basis to understand how much oviposition on treated surfaces is reduced per unit (mg/mL) increase in extract application, with the lowest numbers being the most effective at reducing oviposition (Table 3). Rates of oviposition reduction differed between the species tested. Similar to the EC50 data, for both *B*. *tabaci* (MEAM1) and *F*. *occidentalis*, the most effective extracts were those from LA2560 and LA1376, respectively, while *F*. *fusca* data again revealed the general sensitivity to all extracts tested. Analysis of the proportional reduction in single eggs vs. clustered eggs revealed the differential increases in clustering of oviposited whitefly eggs with the highest amount of clustering observed in eggs laid in response to extracts from *S*. *pennellii* LA1732. The differential clustering of oviposited eggs in response to the different extracts emphasizes the importance of the ROSPF metric that takes into account both egg numbers and clusters of eggs. These results indicate that acylsugars exhibit differential functionality in suppressing oviposition of different insect species, including two within a single insect genus (*Frankliniella*). It follows that acylsugars could thus mediate plant-enemy interactions in such a way as to affect evolution of host specialization, resistance specificity, and host differentiation or local adaptation. This has broad implications for the role of acylsugars in natural plant-enemy evolution. Moreover, the extensive variation in acylsugar composition that bears on plant enemies differently offers potential for deployment of acylsugars as part of a pest management application of acylsugar deployment.

**Table 3:** Rates of oviposition reduction (with confidence intervals) per unit (mg/mL) increase of acylsugar dose. The analyzed responses of *B*. *tabaci* (MEAM1) were the change in number of oviposition sites per female relative to a control treatment, the rates at which females oviposited (aggregate number of eggs) relative to a control treatment, and the rates at which females oviposited in clusters *vs*. single eggs, all during non-choice leaflet oviposition bioassays. The analyzed response of *F*. *occidentalis* and *F*. *fusca* was the probability of oviposition to a treated synthetic membrane during choice bioassays. All treatments were controlled exposure to purified acylsugar extracts, fractions, or blends listed as Acylsugar Source in the table.

	*B*. *tabaci*			*F*. *occidentalis*	*F*. *fusca*
Acylsugar Source	Proportional reduction in ROSPF^a^ per mg/mL acylsugar, and 95% confidence interval	Proportional reduction in REPF^b^ per mg/mL acylsugar, and 95% confidence interval	Proportional reduction in single eggs *vs*. clustered eggs per mg/mL acylsugar, and 95% confidence interval	Proportional reduction in oviposition per mg/mL acylsugar applied to treated surface, and 95% confidence interval	Proportional reduction in oviposition per mg/mL acylsugar applied to treated surface, and 95% confidence interval
CU071026	0.947 (0.929/ 0.966)	0.952 (0.936 / 0.980)	0.950 (0.943/0.956)	0.946 (0.932/ 0.960)	0.703 (0.676/ 0.731)
LA716	0.932 (0.916 /0.948)	0.904 (0.883 / 0.935)	0.909 (0.904/0.914)	0.827 (0.812/ 0.843)	0.757 (0.729/ 0.786)
LA1732	0.894 (0.870/ 0.918)	0.903 (0.881 / 0.931)	0.901 (0.895/0.908)	0.796 (0.778/ 0.816)	0.720 (0.698/ 0.744)
LA2560	0.905 (0.884/ 0.926)	0.873 (0.851 / 0.901)	0.908 (0.901/0.916)	0.682 (0.657/ 0.708)	0.745 (0.717/ 0.775)
LA1376	0.873 (0.851/ 0.895)	0.944 (0.924 / 0.965)	0.910 (0.901/0.919)	0.698 (0.672/ 0.726)	0.612 (0.575/ 0.651)
Fr-LP	0.924 (0.905/ 0.944)	0.934 (0.917 / 0.964)	0.942 (0.936/0.948)	0.733 (0.701/ 0.767)	NT
Fr-MP	0.979 (0.960/ 0.998)	0.966 (0.958 / 1.002)	0.992 (0.984/1.000)	0.840 (0.811/ 0.870)	NT
Fr-MP/Fr-LP	0.905 (0.880/ 0.932)	0.909 (0.887 / 0.940)	0.913 (0.906/0.919)	0.727 (0.693/ 0.762)	NT

NT = Not Tested

^a^ Oviposition metric ROSPF (relative oviposition sites per female) is a normalized quantity of oviposition sites with lower numbers being more effective at reducing oviposition.

^b^ Oviposition metric REPF (relative eggs per female) is a normalized quantity of eggs with lower numbers being more effective at reducing oviposition.

The comparison of the results between the three insect species studied and their respective experimental systems is focused on commonalities in the general effects of acylsugars on these insects: acylsugars reduce oviposition on substrates on which they are present. The magnitude of their effect differs with specific acylsugar composition and concentration; and that there are differences in responses of related species (*F*. *occidentalis* and *F*. *fusca*) when tested using the same experimental system. The assay methods used were different for whitefly vs thrips, since we used standard methods used previously for these species assays performed. The method used for *B*. *tabaci* (MEAM1) was non-choice, while that used on thrips species were choice assays. No implication on mechanism of acylsugar action is intended or implied. Further testing of these acylsugars, species, and experimental systems will be required to determine the specific mechanisms underlying the ovipositional responses observed in this study.

### Fractionation to isolate component effects, and synergism of components

Synergism, the interaction of compounds such that the overall effect is greater than the sum of the their individual effect, could be an important feature of natural systems of insect control involving secondary metabolites [46–52]. To investigate possible synergistic effects between acylsugars, fraction Fr-LP and Fr-MP were derived from the highly active extract of *S*. *pennellii* LA1376. In the absence of synergistic (or antagonistic) effects between the fractions, the effect of the recombined mixture should be equal to the summed effects of the mixture’s constituent parts. For example, the effect of 6 parts of the 1:1 mixture would be equal to the sum effect of 3 parts Fr-LP plus 3 parts Fr-MP, and the generalization of this additive effect is the null hypothesis to which one of synergism or antagonism is alternative. Furthermore, in the absence of synergistic or antagonistic interactions the effect on oviposition for the mixture, per unit increase in dose, would be equal to the average effect per unit dose of the mixture’s two components. In the presence of either of these interactions, the mixture should elicit a different response than that expected if there are no interactions between components of the mixture, and departure from a result consistent with strictly additive effects is indicative of synergism or antagonism, with mixture effect greater than the sum effects of components indicating synergism, and mixture effect being lesser than the sum effects of components indicating antagonism.

Fraction Fr-LP and Fr-MP of *S*. *pennellii* LA1376 were individually tested for effects on oviposition, and a 1:1 recombination of the fractions was similarly tested. Whiteflies exposed to untreated leaf discs exhibited an average of 17.4 oviposition sites per female. Both the Fr-LP and Fr-MP fractions individually suppressed whitefly oviposition relative to the untreated control, with the Fr-LP fraction causing greater suppression than the Fr-MP fraction (Tables 2 and 3). At a rate of 25 mg/mL (the highest observed), Fr-MP/Fr-LP, the 1:1 mixture of the two fractions reduced the number of oviposition sites per female by 75% to 4.4, while Fr-LP and Fr-MP reduced oviposition sites by only 48 and 26%, respectively. The oviposition response by *F*. *occidentalis* in the dual-choice assay was generally consistent with that by *B*. *tabaci* in that Fr-MP/Fr-LP had the greatest effect on oviposition and that the effect of Fr-LP was stronger than was the effect of Fr-MP (Tables 2 and 3).

**Fig 2:**
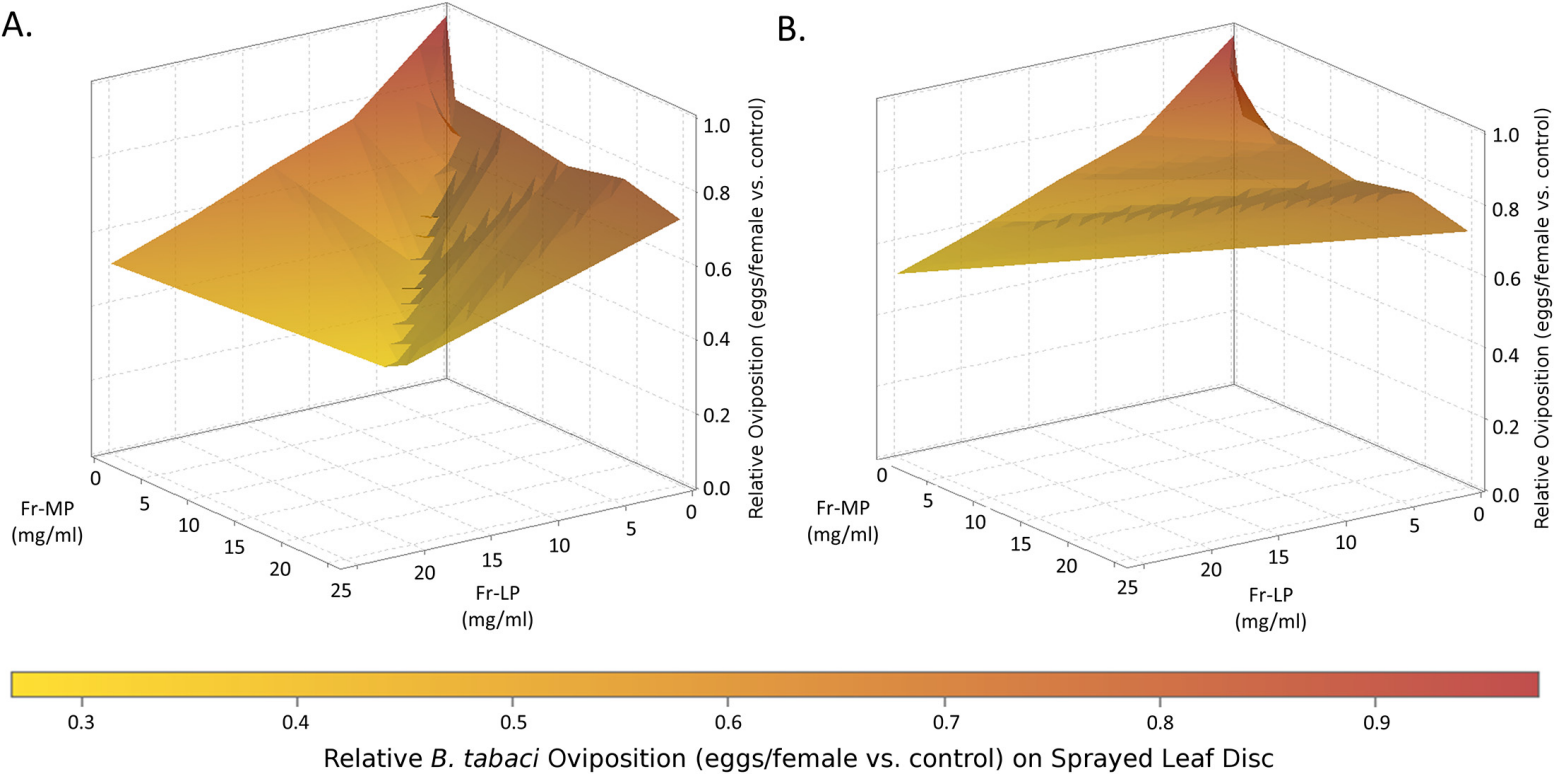
3-D representations of the ovipositional response of *B*. *tabaci* (REPF) to Fr-MP, Fr-LP, and the Fr-MP/Fr-LP blend applied at a range of rates. Panel A shows the actual responses including the synergistic increase in activity of the Fr-MP/Fr-LP blend while panels B shows the actual responses of the two component fractions but with the expected additive response without the synergism of the Fr-MP/Fr-LP blend.

**Fig 3:**
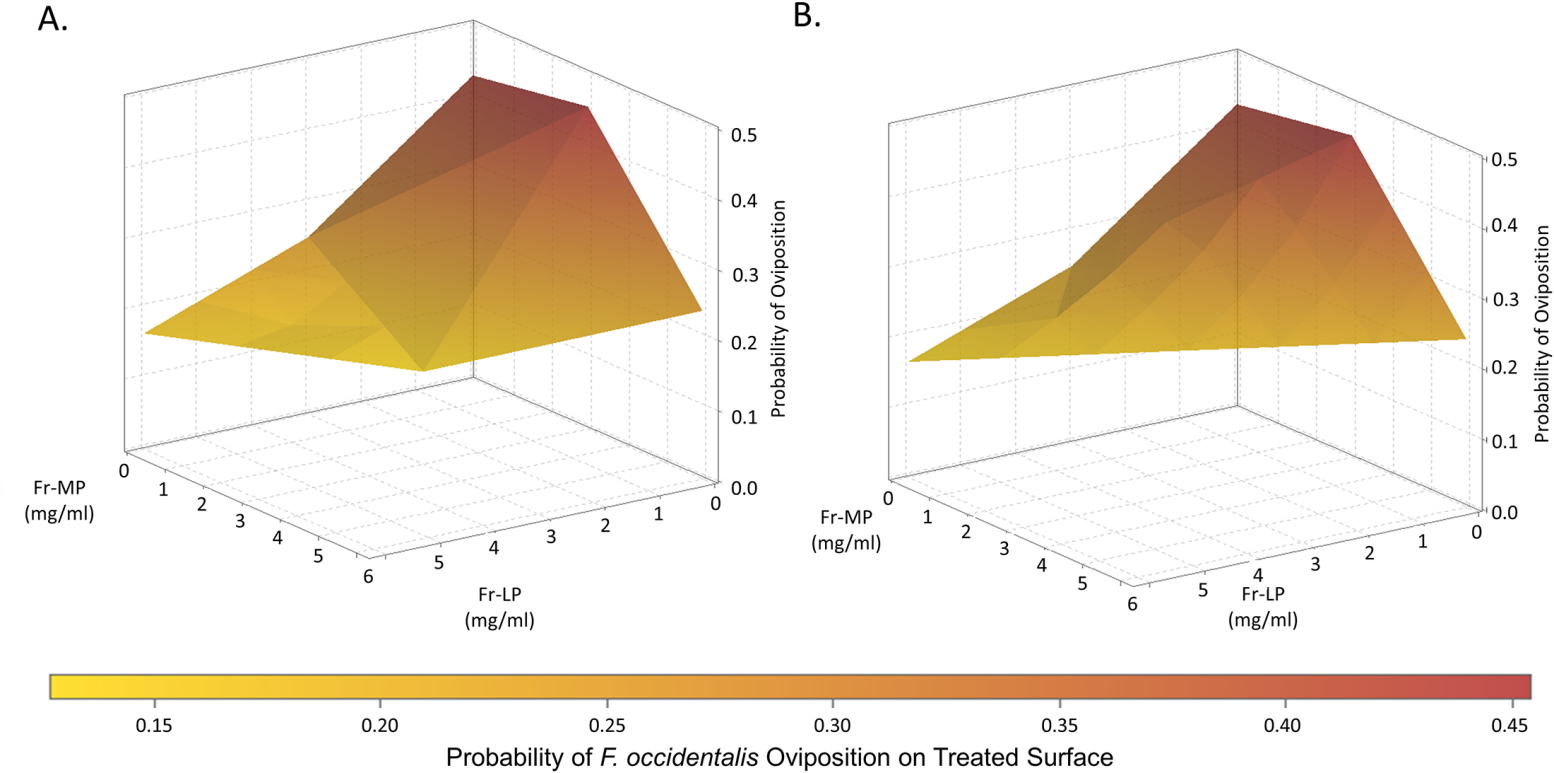
3-D representations of the ovipositional response of *F*. *occidentalis* to Fr-MP, Fr-LP, and the Fr-MP/Fr-LP blend applied at a range of rates. Panel A shows the actual responses including the synergistic increase in activity of the Fr-MP/Fr-LP blend while panels B shows the actual responses of the two component fractions but with the expected additive response without the synergism of the Fr-MP/Fr-LP blend.

The effect of the recombined mixture (Fr-MP/Fr-LP) of the two fractions was stronger than that for the sum of the mixture’s components in both the tests on whiteflies and thrips (Figs 2 and 3). The effects Fr-MP and Fr-LP rate on oviposition were both significant for *B*. *tabaci* (MEAM1) (*p* < 0.002 for each), while only Fr-LP had a significant effect on oviposition by *F*. *occidentalis* (*p* = 0.004). The interaction effect of Fr-MP x Fr-LP rates was significant for both insects (*p* = 0.03 and 0.05, respectively) consistent with the observation that the effect of one fraction depends on the rate of the other fraction—here, the character of that dependence is synergism (Figs 2 and 3). The synergism detected between individual or multiple acylsugars within the two LA1376 fractions has a combined impact that is greater than the sum of the effects of either fraction alone, and is similar to that of the unfractionated acylsugars of *S*. *pennellii* LA1376 (Tables 2 and 3). We believe this to be the first documented case of acylsugar synergism.

In plant-enemy interactions, synergistic effects of compounds lead to a more than additive level of activity. Synergism of defensive secondary metabolites has been identified in multiple classes of plant secondary metabolites including amides and monoterpenoids [49,47,50], and between compound classes [46,48,52]. This can prove adaptive for plants due to increased efficiency of defensive metabolite effects for a given level of production [46,51]. Our experiments suggest observed synergistic effects are predominantly a result of the interaction between different acylsugars (with-in class interaction) rather than their interaction with members of other compound classes (between-class interaction) because the purification procedure used effectively removes most other compounds (S1–S7 Figs). The two acylsugar fractions were separated by a method primarily dependent on differential polarity of the compounds in each fraction, which in this case is largely due to different sugar moieties. However the fatty acids profiles of the two fractions also differ, so it is not possible to attribute the difference in the impact of Fr-MP and Fr-LP, or the synergism when the fractions are combined, to the sugar vs. the fatty acids of the acylsugars in each fraction. We do not yet know which compounds within the fractions mediate this synergism, but these results are a step toward isolating and identifying such compounds.

### Implications of differential impact of acylsugar compositions on its use for insect control

The results of these acylsugar bioassays have major implications for how acylsugar-based insect control strategies can be utilized and improved, both in breeding acylsugar-producing cultivars and in use of synthetic acylsugars as foliar applied crop protectants. The Cornell tomato breeding program developed the line CU071026 and the derived acylsugar-producing lines. Inclusion of additional QTL that increase acylsugar level, either with or without increase of trichome density resulted in lines with stronger control of *B*. *tabaci* (MEAM1) [32], which is consistent with the increases in oviposition suppression with increasing levels of applied acylsugars in the *B*. *tabaci* bioassays. Previous studies have also identified QTL that alter either the acylsugar base moieties or fatty acid profiles in segregating populations [33,34], and shown that transfer of the first of these QTL into CU071026 resulted in the predicted alteration in acylsugar composition [34]. Transfer of the rest of these QTL, as well as additional QTL from both *S*. *pennellii* LA716 or other accessions, should produce a series of tomato breeding lines with varying acylsugar compositions with novel mixtures of base moieties and fatty acids. *In planta* testing of such lines against multiple insect pests would identify the most broadly and strongly resistant lines, facilitating breeding to optimize control of specific pests or pest complexes present in areas in which resistant varieties would be deployed. Since preliminary work suggests that acylsugar-mediated pest resistance could also impact infection of plants by insect-vectored viruses [53], use of tomato lines producing different compositions and/or levels of acylsugars would also provide tools for field applications to control both insect vectors and the viruses they transmit for sustainable crop protection. As plant defenses sometimes come at a fitness cost to plants [54–56], breeding tomato producing different levels and compositions of acylsugars would both provide a platform for testing for fitness costs, and also facilitate optimization of the compositions/acylsugar levels for the control of key insect pests. Utilizing synergism of acylsugar compositions might also lower the levels of acylsugar required for pest control, and thereby lower the potential fitness costs with a concomitant increase in plant vigor and/or yield.

Synthetic acylsugars are also used as a foliar spray for insect control and were developed as contact biocides [57,58]. The optimization of acylsugar sprays evaluated as non-lethal suppressors of insect feeding and oviposition, rather than contact biocides, might be beneficial in that non-lethal methods of control are thought to impose less selective pressure on polyphagous pest species, at least when alternative host plants are available [59,60]. Optimization of acylsugar sprays for non-lethal control might be improved by the blending of multiple acylsugar chemistries to take advantage of synergistic effects. Based on our findings, the utilization of synergistic acylsugar chemistries could significantly increase the efficacy of the acylsugar blend, and possibly reduce the levels needed to achieve effective control. We note that natural *S*. *pennellii* chemistries appear to take advantage of synergistic effects of components, and that this is in turn could offer advantage to applied pest management approaches.

## Supporting Information

S1 Fig Average impact of column purification on level of background compounds within acylsugar extracts before and after purification. (DOCX)

S2 Fig Average impact of column purification on number of background compounds within acylsugar extracts before and after purification. (DOCX)

S3 Fig Average impact of column purification on level of background caffeic acid derivatives within acylsugar extracts before and after purification. (DOCX)

S4 Fig Average impact of column purification on number of background caffeic acid derivatives within acylsugar extracts before and after purification. (DOCX)

S5 Fig Average impact of column purification on level of background flavonoids within acylsugar extracts before and after purification. (DOCX)

S6 Fig Average impact of column purification on number of background flavonoids within acylsugar extracts before and after purification. (DOCX)

S7 Fig HPLC chromatograms of acylsugar extracts A) unpurified CU071026, B) purified CU071026, C) unpurified *S*. *pennellii* LA716, D) purified *S*. *pennellii* LA716, E) unpurified *S*. *pennellii* LA1732, F) purified *S*. *pennellii* LA1732, G) unpurified *S*. *pennellii* LA2560, H) purified *S*. *pennellii* LA2560, I) unpurified *S*. *pennellii* LA1376, J) purified *S*. *pennellii* LA1376, K) *S*. *pennellii* LA1376 fraction Fr-1, L) *S*. *pennellii* LA1376 fraction Fr-2, and M) Fr-MP/Fr-LP.
(DOCX)

S8 Fig Eggs per living female laid by whiteflies presented leaf discs treated with increasing rates of different acylsugar extracts. (DOCX)

S1 Table Oviposition sites per female, eggs per female, percent female mortality, and average number of females per bioassay arena for whiteflies exposed to leaf discs sprayed with *S*. *pennellii* and CU071026 extracts at differing rates. (DOCX)

S2 Table Average eggs oviposited per female on each side in the assay chamber for *F*. *occidentalis* exposed to Parafilm membranes sprayed with *S*. *pennellii* and CU071026 extracts at differing rates. (DOCX)

S3 Table Average eggs oviposited per female on each side in the assay chamber for *F*. *fusca* exposed to Parafilm membranes sprayed with *S*. *pennellii* and CU071026 extracts at differing rates. (DOCX)

S1 Text Method for background phenolics characterization in acylsugar samples. (DOCX)

## References

[1] Wink M. Functions and Biotechnology of Plant Secondary Metabolites. Oxford, UK: Wiley-Blackwell; 2010.

[2] Dixon R. Natural products and plant disease resistance. Nature. 2001;411: 843–847. 11459067 10.1038/35081178

[3] Zhao N, Wang G, Norris A, Chen X, Chen F. Studying Plant Secondary Metabolism in the Age of Genomics. CRC Crit Rev Plant Sci. 2013;32: 369–382.

[4] Trumble JT, Dercks W, Quiros CF, Beier RC. Host plant resistance and linear furanocoumarin content of Apium accessions. J Econ Entomol. 1990;83: 519–525. 2345223 10.1093/jee/83.2.519

[5] Rosenthal JP, Dirzo R. Effects of life history, domestication and agronomic selection on plant defence against insects: Evidence from maizes and wild relatives. Evol Ecol. 1997;11: 337–355.

[6] Wink M. Plant breeding: importance of plant secondary metabolites for protection against pathogens and herbivores. Theor Appl Genet. 1988;75: 225–233.

[7] Juvik J, Stevens M, Rick C. Survey of the genus *Lycopersicon* for variability in alpha-tomatine content. HortScience. 1982;17: 764–766.

[8] Blanca J, Cañizares J, Cordero L, Pascual L, Diez MJ, Nuez F. Variation revealed by SNP genotyping and morphology provides insight into the origin of the tomato. PLoS One. 2012;7: e48198 10.1371/journal.pone.0048198 23118951 PMC3485194

[9] Lange W, Bronson L. Insect pests of tomatoes. Annu Rev Entomol. 1981;26: 345–371.

[10] Schuster DJ, Stansly PA, Polston JE. Expressions of plant damage by *Bemisia* In: *Bemisia*: 1995, taxonomy, biology, damage, control and management. Intercept; 1996 pp. 153–165

[11] Navas-Castillo J, Sánchez-Campos S. Tomato yellow leaf curl virus-Is causes a novel disease of common bean and severe epidemics in tomato in Spain. Plant Dis. 1999;83: 29–32. 10.1094/PDIS.1999.83.1.29 30845435

[12] Polston J, McGovern R, Brown L. Introduction of tomato yellow leaf curl virus in Florida and implications for the spread of this and other geminiviruses of tomato. Plant Dis. 1999;83: 984–988. 10.1094/PDIS.1999.83.11.984 30841296

[13] Polston JE, Anderson PK. The Emergence Of Whitefly-Transmitted Geminiviruses in Tomato in the Western Hemisphere. Plant Dis. The American Phytopathological Society; 1997;81: 1358–1369. 10.1094/PDIS.1997.81.12.1358 30861786

[14] Buta GJ, Lusby WR, Neal JW Jr, Waters RM, Pittarelli GW. Sucrose esters from Nicotiana gossei active against the greenhouse whitefly Trialeuroides vaporarium. Phytochemistry. 1993;32: 859–864.

[15] Cutler HG, Severson RF, Cole PD, Jackson DM, Johnson AW. Secondary metabolites from higher plants: Their possible role as biological control agents In: Natural Resistance of Plants to Pests: Roles of Allelochemicals. Washington, DC: American Chemical Society; 1986 pp. 178–196.

[16] Gibson RW. Trapping of the spider miteTetranychus urticae by glandular hairs on the wild potato *Solanum berthaultii*. Potato Res. 1976;19: 179–182.

[17] Gibson RW, Valencia L. A survey of potato species for resistance to the mite *Polyphagotarsonemus latus*, with particular reference to the protection of *Solanum berthaultii* and *S*. *tarijense* by glandular hairs. Potato Res. 1978;21: 217–223.

[18] Goffreda JC, Mutschler MA. Inheritance of potato aphid resistance in hybrids between *Lycopersicon esculentum* and *L*. *pennellii*. Theor Appl Genet. 1989;78: 210–216. 10.1007/BF00288801 24227146

[19] Goffreda JC. Mutschler MA, Tingey WM. Feeding behavior of potato aphid affected by glandular trichomes of wild tomato. Entomol Exp Appl. 1988;48: 101–107.

[20] Hawthorne DJ, Shapiro JA, Tingey WM, Mutschler MA. Trichome-borne and artificially applied acylsugars of wild tomato deter feeding and oviposition of the leafminer *Liriomyza trifolii*. Entomol Exp Appl. 1992;65: 65–73.

[21] Holley JD, King RR, Singh RP. Glandular trichomes and the resistance of *Solanum berthaultii* (PI 473340) to infection from Phytophthora infestans. Can J Plant Pathol. 1987;9: 291–294.

[22] Juvik JA, Shapiro JA, Young TE, Mutschler MA. Acylglucoses from wild tomatoes alter behavior and reduce growth and survival of *Helicoverpa zea* and *Spodoptera exigua* (Lepidoptera: Noctuidae). J Econ Entomol. 1994;87: 482–492.

[23] Kennedy BS, Nielsen MT, Severson RF, Sisson VA, Stephenson MK, Jackson DM. Leaf surface chemicals from Nicotiana affecting germination of *Peronospora tabacina* (adam) sporangia. J Chem Ecol. 1992;18: 1467–1479. 10.1007/BF00993221 24254279

[24] Liedl BE, Lawson DM, White KK, Shapiro JA, Cohen DE, Carson WG, et al Acylglucoses of the wild tomato *Lycopersicon pennellii* alters settling and reduces oviposition of *Bemisia argentifolii*. J Econ Entomol. 1995;88: 742–748.

[25] Neal JJ, Tingey WM, Steffens JC. Sucrose esters of carboxylic acids in glandular trichomes of *Solanum berthaultii* deter settling and probing by green peach aphid. J Chem Ecol. 1990;16: 487–497. 10.1007/BF01021780 24263505

[26] Neal JJ, Steffens JC, Tingey WM. Glandular trichomes of *Solatium berthaultii* and resistance to the Colorado potato beetle. Entomol Exp Appl. 1989;51: 133–140.

[27] Rodriguez AE, Tingey WM, Mutschler MA. Acylsugars produced by type IV trichomes of *Lycopersicon pennellii* (Corr.)D’Arcy deter settling of the green peach aphid, *Myzus persicae* (Sulzer) (Homoptera: Aphididae). J Econ Entomol. 1993 34–39.

[28] Severson RF, Johnson AW, Jackson DM. Cuticular constituents of tobacco: factors affecting their production and their role in insect and disease resistance and smoke quality. Recent Adv Tob Sci. 1985;11: 105–174.

[29] Burke BA, Goldsby G, Mudd JB. Polar epicuticular lipids of *Lycopersicon pennellii*. Phytochemistry. 1987;26: 2567–2571.

[30] Shapiro JA, Steffens JC, Mutschler MA. Acylsugars of the wild tomato *Lycopersicon pennellii* in relation to geographic distribution of the species. Biochem Syst Ecol. 1994;22: 545–561.

[31] Garrido E, Andraca-Gómez G, Fornoni J. Local adaptation: Simultaneously considering herbivores and their host plants. New Phytol. 2012;193: 445–453. 10.1111/j.1469-8137.2011.03923.x 21988566

[32] Leckie BM, DeJong DM, Mutschler MA. Quantitative trait loci increasing acylsugars in tomato breeding lines and their impacts on silverleaf whiteflies. Mol Breed. 2012;30: 1621–1634.

[33] Leckie BM, DeJong DM, Mutschler MA. Quantitative trait loci regulating sugar moiety of acylsugars in tomato. Mol Breed. 2013;31: 957–970.

[34] Leckie BM, Halitschke R, DeJong DM, Smeda JR, Kessler A, Mutschler MA. Quantitative trait loci regulating the fatty acid profile of acylsugars in tomato. Mol Breed. 2014;34: 1201–1213.

[35] Blauth SL, Steffens JC, Churchill GA, Mutschler MA. Identification of QTLs controlling acylsugar fatty acid composition in an intraspecific population of *Lycopersicon pennellii* (Corr.) D’Arcy. Theor Appl Genet. 1999;99: 373–381.

[36] Blauth SL, Churchill GA, Mutschler MA. Identification of quantitative trait loci associated with acylsugar accumulation using intraspecific populations of the wild tomato, *Lycopersicon pennellii*. Theor Appl Genet. 1998;96: 458–467. 10.1007/s001220050762 24710885

[37] Pedersen DS, Rosenbohm C. Dry Column Vacuum Chromatography. Synthesis (Stuttg). 2001;16: 2431–2434.

[38] Shatters RG, Powell CA, Boykin LM, Liansheng H, McKenzie CL. Improved DNA barcoding method for Bemisia tabaci and related Aleyrodidae: development of universal and Bemisia tabaci biotype-specific mitochondrial cytochrome c oxidase I polymerase chain reaction primers. J Econ Entomol. 2009;102: 750–758. 19449657 10.1603/029.102.0236

[39] McKenzie CL, Bethke JA, Byrne FJ, Chamberlin JR, Dennehy TJ, Dickey AM, et al Distribution of Bemisia tabaci (Hemiptera: Aleyrodidae) Biotypes in North America After the Q Invasion. J Econ Entomol. 2012;105: 753–766. 22812110 10.1603/ec11337

[40] Dinsdale A, Cook L, Riginos C, Buckley YM, De Barro P. Refined Global Analysis of Bemisia tabaci (Hemiptera: Sternorrhyncha: Aleyrodoidea: Aleyrodidae) Mitochondrial Cytochrome Oxidase 1 to Identify Species Level Genetic Boundaries. Ann Entomol Soc Am. 2010;103: 196–208.

[41] Mckenzie CL, Hodges G, Osborne LS; Byrne FJ; Shatters RG. Distribution of Bemisia tabaci (Hemiptera: Aleyrodidae) Biotypes in Florida Investigating the Q Invasion. J Econ Entomol. 2009;102: 670–676. 19449648 10.1603/029.102.0227

[42] Gardner RG, Panthee DR. Tomato Spotted Wilt Virus-resistant Fresh-market Tomato Breeding Lines: NC 58S, NC 123S, NC 127S, and NC 132S. HortScience. 2012;47: 531–532.

[43] Firdaus S, Heusden AW, Hidayati N, Supena EDJ, Visser RGF, Vosman B. Resistance to *Bemisia tabaci* in tomato wild relatives. Euphytica. 2012;187: 31–45.

[44] Whittaker MS, Kirk WDJ. The effects of sucrose and tannin on oviposition by the western flower thrips. Acta Phytopathol Entomol Hungarica. 2004;39: 115–121.

[45] Schilmiller A, Shi F, Kim J, Charbonneau AL, Holmes D, Jones AD, et al Mass spectrometry screening reveals widespread diversity in trichome specialized metabolites of tomato chromosomal substitution lines. Plant J. 2010;62: 391–403. 10.1111/j.1365-313X.2010.04154.x 20113441 PMC2881305

[46] Berenbaum M, Neal JJ. Synergism between myristicin and xanthotoxin, a naturally cooccurring plant toxicant. J Chem Ecol 1985;11: 1349–1358. 10.1007/BF01012136 24311178

[47] Dyer LA, Dodson CD, Stireman JO, Tobler MA, Smilanich AM, Fincher RM, et al Synergistic effects of three Piper amides on generalist and specialist herbivores. J Chem Ecol. 2003;29: 2499–2514. 14682530 10.1023/a:1026310001958

[48] Huang Z, Zhou FC, Xu D, Afzal M, Bashir MH, Ali S, et al Antifeedant activities of secondary metabolites from *Ajuga nipponensis* against *Plutella xylostella*. 2008;40: 1983–1992.

[49] Hummelbrunner LA, Isman MB. Acute, sublethal, antifeedant, and synergistic effects of monoterpenoid essential oil compounds on the tobacco cutworm, Spodoptera litura (Lep., Noctuidae). J Agric Food Chem. 2001;49: 715–720. 11262018 10.1021/jf000749t

[50] Koul O, Singh R, Kaur B, Kanda D. Comparative study on the behavioral response and acute toxicity of some essential oil compounds and their binary mixtures to larvae of *Helicoverpa armigera*, *Spodoptera litura* and *Chilo partellus*. Ind Crops Prod. 2013;49: 428–436.

[51] Mckey D. The distribution of secondary compounds within plants In: Rosenthal G. A.; Jansen DH, editor. Herbivores: their interactions with secondary plant metabolites. Academic Press; 1979 pp. 55–133.

[52] Steppuhn A, Baldwin IT. Resistance management in a native plant: nicotine prevents herbivores from compensating for plant protease inhibitors. Ecol Lett. 2007;10: 499–511. 17498149 10.1111/j.1461-0248.2007.01045.x

[53] Mutschler MA, Wintermantel WM. Reducing virus associated crop loss through resistance to insect vectors In: Natural Resistance Mechanisms of Plants to Viruses. Springer; 2006 pp. 241–260.

[54] Bergelson J, Purrington CB. Surveying patterns in the cost of resistance in plants. Am Nat. 1996;148: 536–558.

[55] Elle E, van Dam NM, Hare JD. Cost of glandular trichomes, a “resistance” character in *Datura wrightii* Regel (Solanaceae). Evolution. 1999;53: 22–35. 28565189 10.1111/j.1558-5646.1999.tb05330.x

[56] Hare JD, Elle E, van Dam NM. Costs of glandular trichomes in *Datura wrightii*: a three-year study. Evolution. 2003;57: 793–805. 12778549 10.1111/j.0014-3820.2003.tb00291.x

[57] Chortyk OT, Kays SJ, Teng Q. Characterization of Insecticidal Sugar Esters of Petunia. J Agric Food Chem. 1997;45: 270–275.

[58] Puterka GJ, Farone W, Palmer T, Barrington A. Structure-function relationships affecting the insecticidal and miticidal activity of sugar esters. J Econ Entomol. 2003;96: 636–44. 12852599 10.1603/0022-0493-96.3.636

[59] Gould F. Arthropod Behavior And The Efficacy Of Plant Protectants. Annu Rev of Entomol. 1991 pp. 305–330.

[60] Gould F. Role of behavior in the evolution of insect adaptation to insecticides and resistant host plants. Bull ESA. 1984; 34–41.

