# Supplementary material for: Differential and Synergistic Functionality of Acylsugars in Suppressing Oviposition by Insect Herbivores
Source: PLoS One. 2016 Apr 11;11(4):e0153345. doi: 10.1371/journal.pone.0153345 (PMC4827819; doi:10.1371/journal.pone.0153345)
Supplement: S5 Fig — (DOCX) [file pone.0153345.s005.docx]

S5 Fig. Average impact of column purification on level of background flavonoids within acylsugar extracts before and after purification. Single samples of each bulked acylsugar extract from the accessions/breeding lines were analyzed by HPLC to provide these average background levels before and after purification. Acylsugars are not visualized by HPLC and are not included in this analysis. Error Bars = Standard deviation.
