# Supplementary material for: Differential and Synergistic Functionality of Acylsugars in Suppressing Oviposition by Insect Herbivores
Source: PLoS One. 2016 Apr 11;11(4):e0153345. doi: 10.1371/journal.pone.0153345 (PMC4827819; doi:10.1371/journal.pone.0153345)
Supplement: S7 Fig — A) unpurified CU071026, B) purified CU071026, C) unpurified S. pennellii LA716, D) purified S. pennellii LA716, E) unpurified S. pennellii LA1732, F) purified S. pennellii LA1732, G) unpurified S. pennellii LA2560, H) purified S. pennellii LA2560, I) unpurified S. pennellii LA1376, J) purified S. pennellii LA1376, K) S. pennellii LA1376 fraction Fr-1, L) S. pennellii LA1376 fraction Fr-2, and M) Fr-MP/Fr-LP. (DOCX) [file pone.0153345.s007.docx]

A.

UV Absorption at 320nm

Retention Tin

Retention time (min)

B.

UV Absorption at 320nm

Retention time (min)

C.

UV Absorption at 320nm

Retention time (min)

D.

UV Absorption at 320nm

Retention time (min)

E.

UV Absorption at 320nm

Retention time (min)

F.

UV Absorption at 320nm

Retention time (min)

G.

UV Absorption at 320nm

Retention time (min)

H.

UV Absorption at 320nm

Retention time (min)

I.

UV Absorption at 320nm

Retention time (min)

J.

UV Absorption at 320nm

Retention time (min)

K.

UV Absorption at 320nm

Retention time (min)

L.

UV Absorption at 320nm

Retention time (min)

M.

UV Absorption at 320nm

Retention time (min)

S7 Fig. HPLC chromatograms of acylsugar extracts A) unpurified CU071026, B) purified CU071026, C) unpurified *S. pennellii* LA716, D) purified *S. pennellii* LA716, E) unpurified *S. pennellii* LA1732, F) purified *S. pennellii* LA1732, G) unpurified *S. pennellii* LA2560, H) purified *S. pennellii* LA2560, I) unpurified *S. pennellii* LA1376, J) purified *S. pennellii* LA1376, K) *S. pennellii* LA1376 fraction Fr-1, L) *S. pennellii* LA1376 fraction Fr-2, and M) Fr-MP/Fr-LP.
