# Supplementary material for: Differential and Synergistic Functionality of Acylsugars in Suppressing Oviposition by Insect Herbivores
Source: PLoS One. 2016 Apr 11;11(4):e0153345. doi: 10.1371/journal.pone.0153345 (PMC4827819; doi:10.1371/journal.pone.0153345)
Supplement: S8 Fig — (DOCX) [file pone.0153345.s008.docx]

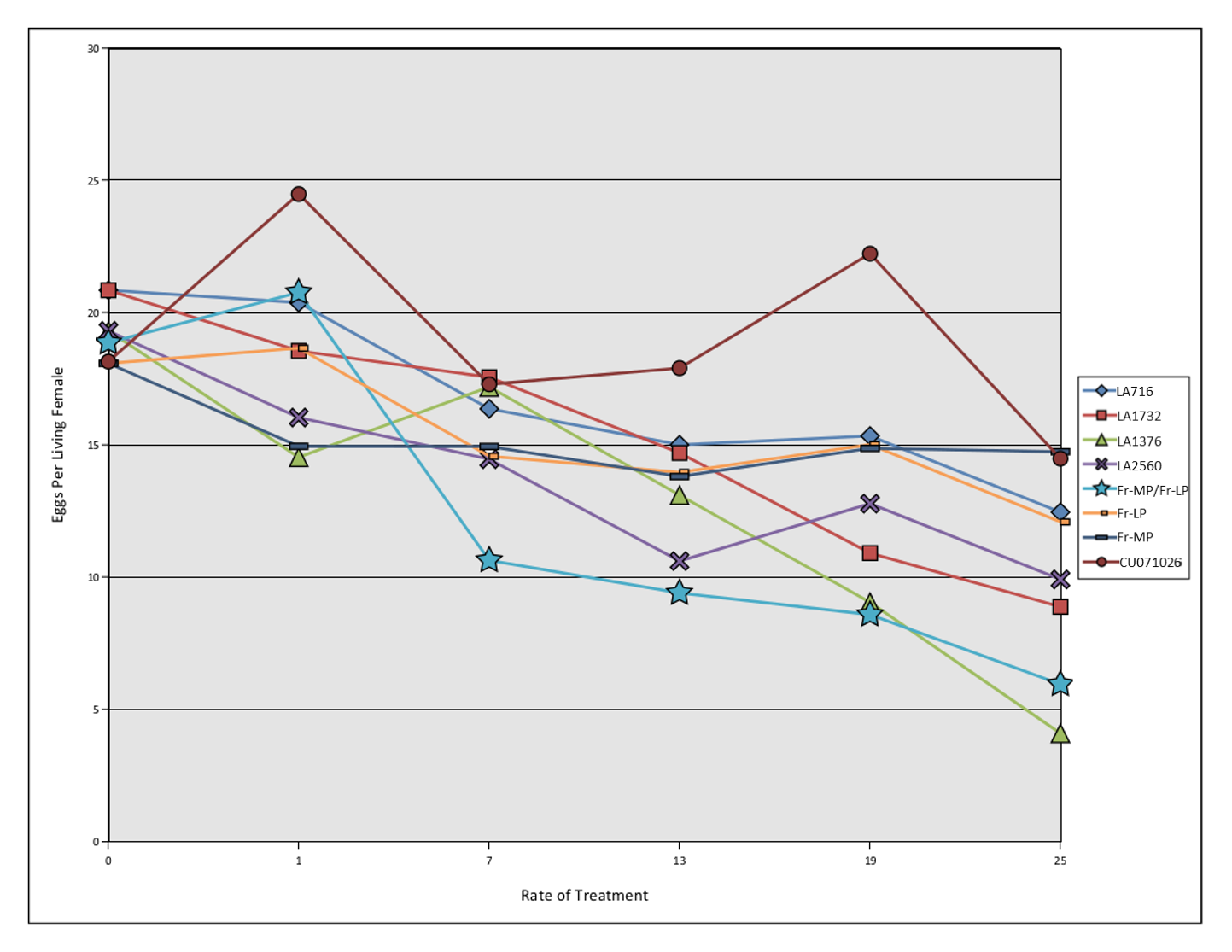


S8 Fig. Eggs per living female laid by whiteflies presented leaf discs treated with increasing rates of different acylsugar extracts.
