# Supplementary material for: Differential and Synergistic Functionality of Acylsugars in Suppressing Oviposition by Insect Herbivores
Source: PLoS One. 2016 Apr 11;11(4):e0153345. doi: 10.1371/journal.pone.0153345 (PMC4827819; doi:10.1371/journal.pone.0153345)
Supplement: S1 Table — (DOCX) [file pone.0153345.s009.docx]

S1 Table. Oviposition sites per female, eggs per female, percent female mortality, and average number of females per bioassay arena for whiteflies exposed to leaf discs sprayed with *S. pennellii* and CU071026 extracts at differing rates.

| Source of extract | Rate of extracts applied (mg/ml) | | | | | | | | | | | | | | | | | | | | | | | |
| --- | --- | --- | --- | --- | --- | --- | --- | --- | --- | --- | --- | --- | --- | --- | --- | --- | --- | --- | --- | --- | --- | --- | --- | --- |
|  | 0 | | | | 1 | | | | 7 | | | | 13 | | | | 19 | | | | 25 | | | |
|  | OSPF^a^ | EPF^b^ | PFM^c^ | AF^d^ | OSPF | EPF | PFM | AF | OSPF | EPF | PFM | AF | OSPF | EPF | PFM | AF | OSPF | EPF | PFM | AF | OSPF | EPF | PFM | AF |
| EtOH Control^e^ | 18.9 | 19.1 | 5.3 | 6.2 | - | - | - |  | - | - | - |  | - | - | - |  | - | - | - |  | - | - | - |  |
| LA716 | - | - | - |  | 17.0 | 17.3 | 6.9 | 7.4 | 13.3 | 14.9 | 18.3 | 11.0 | 11.4 | 13.4 | 16.6 | 7.9 | 10.5 | 13.6 | 16.6 | 8.3 | 8.3 | 10.7 | 16.7 | 8.2 |
| LA1732 | - | - | - |  | 17.1 | 17.5 | 13.8 | 7.3 | 13.7 | 14.6 | 18.0 | 9.2 | 9.9 | 11.9 | 23.2 | 11.8 | 7.3 | 9.1 | 23.6 | 10.2 | 4.4 | 6.0 | 39.8 | 7.0 |
| LA1376 | - | - | - |  | 15.4 | 15.6 | 7.0 | 6.8 | 11.1 | 12.4 | 34.4 | 7.8 | 6.8 | 8.9 | 29.9 | 9.8 | 5.0 | 6.2 | 20.4 | 7.7 | 2.9 | 3.6 | 21.4 | 6.8 |
| LA2560 | - | - | - |  | 14.6 | 15.2 | 13.3 | 6.7 | 11.9 | 12.9 | 12.2 | 7.3 | 6.5 | 7.7 | 16.5 | 6.7 | 6.7 | 9.0 | 37.2 | 7.7 | 5.0 | 6.0 | 53.9 | 8.2 |
| CU071026 | - | - | - |  | 17.1 | 17.6 | 13.9 | 5.2 | 14.9 | 15.6 | 14.5 | 9.2 | 16.2 | 17.8 | 27.2 | 6.2 | 13.4 | 14.7 | 30.6 | 8.2 | 9.9 | 11.4 | 31.6 | 9.3 |
| EtOH Control^f^ | 17.4 | 17.7 | 5.1 | 10.4 | - | - | - |  | - | - | - |  | - | - | - |  | - | - | - |  | - | - | - |  |
| Fr-MP/Fr-LP | - | - | - |  | 16.8 | 17.3 | 6.5 | 12.0 | 9.1 | 10.1 | 8.8 | 10.8 | 7.2 | 9.0 | 7.1 | 11.8 | 6.3 | 8.1 | 11.6 | 14.0 | 4.4 | 5.6 | 10.7 | 11.7 |
| Fr-LP | - | - | - |  | 16.9 | 17.1 | 12.8 | 9.7 | 13.5 | 14.1 | 2.4 | 10.7 | 12.1 | 12.9 | 7.3 | 10.7 | 10.5 | 11.8 | 25.0 | 11.5 | 9.1 | 10.9 | 18.2 | 10.2 |
| Fr-MP | - | - | - |  | 13.7 | 14.1 | 9.6 | 10.0 | 13.4 | 13.9 | 11.8 | 10.2 | 12.9 | 13.2 | 6.8 | 9.7 | 13.5 | 14.1 | 8.9 | 9.2 | 12.8 | 13.3 | 13.5 | 12.3 |

^a^ Oviposition sites per female

^b^ Eggs per female

^c^ Percent female mortality

^d^ Average number of females per bioassay arena

^e^ Ethanol control for initial experiments

^f^ Ethanol control for fractionation experiments
