# Supplementary material for: Differential and Synergistic Functionality of Acylsugars in Suppressing Oviposition by Insect Herbivores
Source: PLoS One. 2016 Apr 11;11(4):e0153345. doi: 10.1371/journal.pone.0153345 (PMC4827819; doi:10.1371/journal.pone.0153345)
Supplement: S2 Table — (DOCX) [file pone.0153345.s010.docx]

S2 Table. Average eggs oviposited per female on each side in the assay chamber for *F. occidentalis* exposed to Parafilm membranes sprayed with *S. pennellii* and CU071026 extracts at differing rates.

| Source of extract | Rate of extracts applied (mg/ml) | | | | | | | | | | | |
| --- | --- | --- | --- | --- | --- | --- | --- | --- | --- | --- | --- | --- |
|  | 0 | | 1 | | 5 | | 10 | | 15 | | 20 | |
|  | UN^a^ | SPR^b^ | UN | SPR | UN | SPR | UN | SPR | UN | SPR | UN | SPR |
| LA716 | 5.6 | 5.3 | 7.1 | 6.7 | 8.4 | 2.9 | 11.8 | 1.9 | 8.3 | 0.0 | 8.0 | 0.0 |
| LA1732 | 5.8 | 4.6 | 10.0 | 4.4 | 8.0 | 3.3 | 12.9 | 1.6 | 11.3 | 0.1 | 12.8 | 0.0 |
| LA1376 | 8.9 | 4.8 | 7.0 | 5.9 | 10.7 | 1.6 | 14.2 | 0.0 | 9.0 | 0.0 | 13.8 | 0.0 |
| LA2560 | 12.5 | 5.8 | 7.1 | 3.8 | 15.4 | 2.6 | 18.5 | 1.3 | 10.3 | 0.5 | 16.3 | 0.0 |
| CU071026 | 3.3 | 5.3 | 6.9 | 3.6 | 3.8 | 3.7 | 7.1 | 0.9 | 8.7 | 2.4 | 5.5 | 3.9 |
| Fr-LP | 8.2 | 9.1 | 9.2 | 8.6 | 9.9 | 4.4 | 15.7 | 1.3 | 13.9 | 0.5 | 14.8 | 0.0 |
| Fr-MP | 7.5 | 5.6 | 7.2 | 8.8 | 11.8 | 9.4 | 10.5 | 8.9 | 11.3 | 7.3 | 8.3 | 6.2 |

^a^ Unsprayed side

^b^ Sprayed side
