# Supplementary material for: Differential and Synergistic Functionality of Acylsugars in Suppressing Oviposition by Insect Herbivores
Source: PLoS One. 2016 Apr 11;11(4):e0153345. doi: 10.1371/journal.pone.0153345 (PMC4827819; doi:10.1371/journal.pone.0153345)
Supplement: S3 Table — (DOCX) [file pone.0153345.s011.docx]

S3 Table. Average eggs oviposited per female on each side in the assay chamber for *F. fusca* exposed to Parafilm membranes sprayed with *S. pennellii* and CU071026 extracts at differing rates.

| Source of extract | Rate of extracts applied (mg/ml) | | | | | | | | | | | |
| --- | --- | --- | --- | --- | --- | --- | --- | --- | --- | --- | --- | --- |
|  | 0 | | 1 | | 5 | | 10 | | 15 | | 20 | |
|  | UN^a^ | SPR^b^ | UN | SPR | UN | SPR | UN | SPR | UN | SPR | UN | SPR |
| LA716 | 4.9 | 3.7 | 5.6 | 4.4 | 6.8 | 2.4 | 7.4 | 0.1 | 11.8 | 0.5 | 9.2 | 0.0 |
| LA1732 | 5.5 | 5.8 | 4.1 | 3.9 | 7.2 | 0.6 | 5.9 | 0.8 | 7.8 | 0.3 | 6.3 | 0.1 |
| LA1376 | 3.9 | 6.3 | 9.1 | 4.1 | 10.2 | 0.6 | 9.4 | 0.3 | 10.8 | 0.0 | 9.2 | 0.0 |
| LA2560 | 4.5 | 3.3 | 5.1 | 1.8 | 5.0 | 0.6 | 5.6 | 0.0 | 2.9 | 0.0 | 6.8 | 0.9 |
| CU071026 | 5.6 | 4.2 | 7.9 | 3.8 | 7.8 | 0.6 | 6.9 | 0.2 | 10.9 | 0.8 | 9.4 | 0.3 |
| Fr-LP | 5.3 | 5.0 | 5.6 | 2.5 | 9.1 | 1.9 | 7.6 | 0.2 | 8.2 | 0.1 | 12.7 | 0.4 |
| Fr-MP | 7.8 | 5.5 | 7.1 | 6.5 | 9.9 | 3.1 | 8.7 | 4.6 | 8.8 | 5.1 | 7.3 | 5.9 |

^a^ Unsprayed side

^b^ Sprayed side
