# Supplementary material for: Differential and Synergistic Functionality of Acylsugars in Suppressing Oviposition by Insect Herbivores
Source: PLoS One. 2016 Apr 11;11(4):e0153345. doi: 10.1371/journal.pone.0153345 (PMC4827819; doi:10.1371/journal.pone.0153345)
Supplement: S1 Text — (DOCX) [file pone.0153345.s012.docx]

S1 Text. Method for background phenolics characterization in acylsugar samples.

Phenolic compound content of trichome extracts was analyzed before and after purification for quality control and to control for possible impact of plant secondary metabolites other than acyl sugars in mediating insect resistance in the tested plant chemotypes. Phenolic compounds were analyzed out of the extracts by HPLC (Agilent 1100, Santa Clara, CA, USA) on a C18 column (4.6 x 150 mm, 3μm particle size, Gemini, Phenomenex, Torrance, CA, USA) as described by [38]. Each compound was quantified as peak area (signal intensity) relative to the initial tissue amount. Compounds, were classified by comparing peak retention times and UV spectra with those of published spectra and authentic standards.
